# Pharmacological Properties of *Vochysia Haenkeana* (Vochysiaceae) Extract to Neutralize the Neuromuscular Blockade Induced by Bothropstoxin-I (Lys49 Phospholipase A_2_) Myotoxin

**DOI:** 10.15171/apb.2017.052

**Published:** 2017-09-25

**Authors:** Carolina Harder, Akila Lara de Oliveira, Andreia Borges Scriboni, Adélia Cristina Oliveira Cintra, Raphael Schezaro-Ramos, Márcio Galdino dos Santos, Karina Cogo-Müller, Regina Yuri Hashimoto Miura, Rafael Stuani Floriano, Sandro Rostelato-Ferreira, Yoko Oshima-Franco

**Affiliations:** ^1^Institute of Health Sciences, Universidade Paulista (Unip), Av. Independência 210, 18087-100, Sorocaba, SP, Brazil.; ^2^University of Sorocaba (Uniso), Rodovia Raposo Tavares km 92.5, 18023-000, Sorocaba, SP, Brazil.; ^3^Department of Pharmacology, Piracicaba Dental School, State University of Campinas (UNICAMP), Av. Limeira 901, 13414-903, Piracicaba, SP, Brazil.; ^4^Faculty of Pharmaceutical Sciences, Department of Clinical and Toxicological Analysis and Bromatology, São Paulo University (USP), Via do Café S/N, 14040-903, Ribeirão Preto, SP, Brazil.; ^5^Department of Pharmacology, Faculty of Medical Sciences, State University of Campinas (UNICAMP), Rua Tessália Vieira de Camargo 126, 13083-887, Campinas, SP, Brazil.; ^6^Tocantins Federal University (UFT), Av. NS 15, ALC NO14, 109 Norte, 77001-090, Palmas, TO, Brazil.; ^7^Faculty of Pharmaceutical Sciences, State University of Campinas (UNICAMP), Rua Cândido Portinari, 200, 13083-871, Campinas, SP, Brazil.

**Keywords:** Antimicrobial activity, Antiophidian activity, Bothropstoxin-I, Myotoxicity, Neurotoxicity, V. haenkeana extract

## Abstract

***Purpose:*** Bothrops snakes are responsible for more than 70 % of snakebites every year in Brazil and their venoms cause severe local and systemic damages. The pharmacological properties of medicinal plants have been widely investigated in order to discover new alternative treatments for different classes of diseases including neglected tropical diseases as envenomation by snakebites. In this work, we have investigated the ability of Vochysia haenkeana stem barks extract (VhE) to neutralize the neuromuscular effects caused by Bothropstoxin-I (BthTX-I), the major phospholipase A_2_ (PLA_2_) myotoxin from B. jararacussu venom.

***Methods:*** The biological compounds of VhE were analysed under thin layer chromatography (TLC) and its neutralizing ability against BthTX-I was assessed through twitch-tension recordings and histological analysis in mouse phrenic nerve-diaphragm (PND) preparations. The antimicrobial activity of VhE was assessed against S. aureus, E. coli and P. aeruginosa strains. The aggregation activity of VhE was analysed under protein precipitation assay.

***Results:*** VhE showed the presence of phenolic compound visualized by blue trace under TLC. VhE abolished the neuromuscular blockade caused by BthTX-I applying the pre-toxin incubation treatment and partially neutralized the BthTX-I action under post-toxin incubation treatment; VhE contributed slightly to decrease the myotoxicity induced by BthTX-I. The neutralizing mechanism of VhE may be related to protein aggregation. VhE showed no antimicrobial activity.

***Conclusion:*** V. haenkeana extract which has no antimicrobial activity exhibited neutralizing ability against the neuromuscular blockade caused by BthTX-I and also contributed to decrease its myotoxicity. Protein aggregation involving phenolic compounds may be related in these protective effects.

## Introduction

In Brazil, *Bothrops* snakes comprise more than 30 species distributed throughout the country and they are responsible for approximately 70 % of snakebites every year; the World Health Organization (WHO) has considered snakebites as a neglected tropical disease due to the numerous cases and difficulties in specific regions to reach antivenom therapy.^1-5^

*Bothrops* venoms induce severe local and systemic damages due to their high enzymatic action basically mediated by proteases and phospholipases A_2_ (PLA_2_).^6,7^ In envenomation by *Bothrops* venoms, the local effects are marked by intense necrosis accompanied by edema, equimosis and acute inflammatory activity. In addition, local infections caused by gram-negative anaerobic bacteria derived from oral flora of snakes are considered an important clinical complication in victims of snakebites.^4,8^ Snakebites are conventionally treated through antivenom serum therapy; however, based on popular practices, the pharmacological properties of medicinal plants have been widely investigated in order to discover new alternative treatments for different classes of diseases including neglected tropical diseases as envenomation by snakebites.^9^ Recent investigations have shown that plant extracts exhibit antimicrobial and antiophidian activities.^10-12^

*Bothrops jararacussu* snake, popularly known as Jararacuçu, is widely distributed in Southeast region of Brazil^6^ and its venom is composed by enzymatic and non-enzymatic proteins, carbohydrates, peptides, lipids, biogenic amines and inorganic components, similarly to other *Bothrops* venoms.^13^ Bothropstoxin-I (BthTX-I) is a non-enzymatic Lys49 PLA_2_ isolated from *B. jararacussu* venom which induces irreversible neuromuscular blockade in vertebrate neuromuscular preparations *in vitro* characterized by intense myonecrosis, increase in creatine kinase release, muscle contracture and membrane depolarization.^14-17^

*Vochysia haenkeana* (Vochysiaceae), plant popularly known in Brazil as “escorrega-macaco”, “pau-amarelo” and/or “cambarazinho”, comes from semidecidual broadleaf forest common in Mato Grosso do Sul, Mato Grosso and Goiás Brazilian States; *V. haenkeana* exhibits a great variety of secondary metabolites such as tannins, saponins, phenolic compounds, flavonoids and coumarins and it has been cited in ethnobotanical studies related to treatment of respiratory diseases.^18-20^ However, its pharmacological activities have been poorly investigated.^21^

In this work, we have assessed the neutralizing ability of *V. haenkeana* hydroalcoholic extract against the main PLA_2_-myotoxin (BthTX-I) from *B. jararacussu* venom in mouse nerve-diaphragm preparations and its antimicrobial activity against *Staphyloccus aureus*, *Escherichia coli* and *Pseudomonas aeruginosa* strains.

## Materials and Methods

### Reagents and BthTX-I

All salts for the physiological solution were of analytical grade. BthTX-I was provided by Dra Adélia Cristina Oliveira Cintra from São Paulo University (USP, Ribeirão Preto, SP, Brazil).

### Animals

Male Swiss mice (25–30 g) obtained from Multidisciplinary Center for Biological Investigation (CEMIB/Unicamp) were housed at a maximum of 10 mice per cage at 23 °C on a 12 h light/dark cycle. The animals had free access to food and water *ad libitum*.

### Plant material

The hydroalcoholic extract from stem barks of *V. haenkeana* was provided by Dr Márcio Galdino dos Santos from Tocantins Federal University (UFT, Palmas, TO, Brazil). The full description about the origin of the extract has been shown elsewhere.^18^ The plant exsiccate was deposited in the Herbarium of the Tocantins Federal University (UFT, Porto Nacional, TO, Brazil) as voucher specimen #10.074 by Solange de Fátima Lolis according to the International Code of Botanical Nomenclature (ICBN).

### Solubilisation of V. haenkeana extract

In order to find out the ideal solvent for *V. haenkeana* extract (VhE) without affecting the basal twitch responses recorded in mammalian nerve-muscle preparations, polyethylene glycol 400 (PEG 400, Synth®), dimethyl sulfoxide (DMSO, Sigma^®^) and ethanol (Synth^®^) were selected to be tested. Ethanol (30 µL) showed be the best solvent to solubilize VhE; ethanol 70 % did not change the twitch responses in control experiments recorded from mouse phrenic nerve-diaphragm (PND) preparations.^22^

### Thin layer chromatography (TLC)

For TLC, it was used aluminium plates coated with silica gel 60 (0.20 mm thick) containing the fluorescent indicator UV254 (Macherey-Nagel GmbH & Co., Bethlehem, PA, USA) and phytochemical standard in methanol (1 %) (Sigma-Aldrich Co., St. Louis, MO, USA) including quercetin (1), rutin (2), caffeic acid (3), tannic acid (4), coumarin (5), gallic acid (6) and VhE (7); a purposeful empty lane was maintained for observing the solvent race (8). The solvent system (mobile phase, 10 mL) consisted of ethyl acetate, formic acid, acetic acid and water (100:11:11:27), as described elsewhere.^23^ The chromatograms were initially stained with diphenylboric acid 2-aminoethyl ester solution (5 % in ethanol) (Sigma-Aldrich Co., St. Louis, MO, USA) followed by polyethylene glycol 4000 solution (5 % in ethanol) (Sigma-Aldrich Co., St. Louis, MO, USA), with visualization under UV light at 360 nm. The retention factor (*Rf*) and sample colours were visually compared to phytochemical standards.

### Antimicrobial activity of VhE

The following bacterial strains were purchased from American Type Culture Collection (ATCC): *Escherichia coli* ATCC 25922, *E. coli* ATCC 10536, *Pseudomonas aeruginosa* ATCC 25922, *S. aureus* ATCC 29213, methicillin-resistant *S. aureus* (MRSA) ATCC 33591, and methicillin/oxacillin resistant *S. aureus* (MRSA/ORSA) ATCC 43300. The cultures were stored at -80 °C in Tryptic soy broth (TSB; Difco Laboratories, Detroit MI, USA) containing 40 % (v/v) glycerol and were routinely cultured in Tryptic soy agar (TSA; Difco Laboratories) under aerobic conditions at 37 °C.

Antimicrobial activity of the extract was tested by using the broth microdilution method according to the guidelines from the Clinical and Laboratory Standards Institute (CLSI), protocol M07-A9. Briefly, four to five colonies were harvested from pure cultures growing on TSA and were used to prepare a bacterial inoculum. Colonies were transferred into tubes containing 5 mL of TSB and cultured at 37 °C until reaching turbidity equivalent to 0.1 at 660 nm (approximately 1.5 x 10^8^ CFU/mL). Two-fold dilutions of the VhE (from 1000 to 0.4 µg/mL) were made in 96-well plates with 100 µL of Mueller Hinton Broth (MHB; Difco) per well. Then, the bacterial suspension (100 μL) was inoculated, and the plates were incubated for 24 h at 37 °C. The lowest concentration with any visible bacterial growth was taken as the minimum inhibitory concentration (MIC). In addition, bacterial growth was assessed by optical density measurement (660 nm).

### Mouse phrenic nerve-diaphragm (PND) preparation

PND preparations were obtained from male Swiss mice killed with isoflurane (Fortvale^®^, Vinhedo, SP, Brazil). The preparations were mounted under a resting tension of 5 g in a 5 mL organ bath containing aerated (95 % O_2_/5 % CO_2_) Tyrode solution (composition, in mM: NaCl 137, KCl 2.7, CaCl_2_ 1.8, MgCl_2_ 0.49, NaH_2_PO_4_ 0.42, NaHCO_3_ 11.9 and glucose 11.1, pH 7.0) at 37 °C as described elsewhere.^24^ The preparations were stimulated indirectly (5–7 V, 0.1 Hz, 0.2 ms) with supramaximal stimuli being delivered from a stimulator (Model ESF-15D, Ribeirão Preto, SP, Brazil) via bipolar electrodes positioned on the nerve. Muscle twitches were recorded using a force displacement transducer coupled to a two-channel Gemini recorder (both from Ugo Basile^®^, Varese, Italy). After stabilization for 20 min, VhE and BthTX-I (a single concentration per experiment) was added to the preparations and left in contact for 120 min or until complete blockade. For neutralization assays, we applied two types of protocols, as suggested elsewhere:^25^ 1) pre-toxin incubation; BthTX-I was maintained under incubation with VhE for 30 min before twitch tension experiments) and 2) post-toxin incubation (VhE was added into the recording chamber 10 min later than BthTX-I).

### Protein precipitation measurement 

The protein precipitation induced by VhE extract was evaluated as previously described elsewhere.^26,27^ Albumin and BthTX-I (10 μg) was incubated separately with VhE (150 μg) following the protocols: 1) pre-toxin incubation with VhE: BthTX-I (50 mg/mL) was maintained under incubation with VhE (0.4 mg/mL) for 30 min at room temperature (23-25 °C) and then for 60 min at 37 °C; 2) without pre-toxin incubation with VhE: BthTX-I (50 mg/mL) was maintained incubated with VhE (0.4 mg/mL) for 60 min directly at 37 °C. In both of protocols, the mixture was centrifuged at 5,000 rpm for 15 min and the protein concentration in supernatant was measured as essentially described elsewhere.^28^ The absorbance obtained was compared with tubes containing only protein (albumin or BthTX-I) to assess the % of protein precipitation. Ethanol (solvent for VhE) was tested alone to verify its influence on the protein precipitation. Tubes containing the same amount of VhE or ethanol, in absence of albumin or BthTX-I, were used as blanks.

### Quantitative histological study

The preparations from the preincubation and post toxin assays were analysed by a quantitative morphometric method and compared to Tyrode control, VhE and BthTX-I. At the end of each experiment (after 120 min), three preparations of each group were fixed by a formalin 10 % solution, and processed by routine morphological techniques. Cross-sections (5 µm thick) of diaphragm muscle were stained with 0.5 % (w/v) hematoxylin-eosin, for microscopy examination. Tissue damage (edema, intense myonecrosis characterized by atrophy of the muscle fibers, hyaline aspect, sarcolemmal disruption and lysis of the myofibrils) was verified by three different trained people and it was expressed as a myotoxicity index (MI), *i.e*., the percentage of damaged muscle cells number divided by the total number of cells in three non-overlapping, non-adjacent areas of each preparation.^29^

### Statistical analysis

Changes in the twitch-tension responses of PND preparations were expressed as a percentage relative to baseline (time zero) values and morphological alterations measured based on myotoxicity index (MI, in %). The results were expressed as the mean ± SEM and statistical comparisons were done using Student's* t*-test with *p* < 0.05 indicating significance. All data analyses were done using Microcal Origin 8 SR4 v.8.0951 (Microcal Software Inc., Northampton, MA, USA) software.

## Results and Discussion

Based on popular medicinal practices, stem barks from *Vochysia haenkeana* (a plant variety of the Vochysiaceae family widely distributed in semidecidual broadleaf forest from west central region of Brazil) has been selected in this work to be investigated in terms of its neutralizing ability against the effects induced by snake venoms and antimicrobial activity. *V. haenkeana* is popularly known as “escorrega-macaco” and shows long plump trunk and smooth barks.

**Figure 1:**
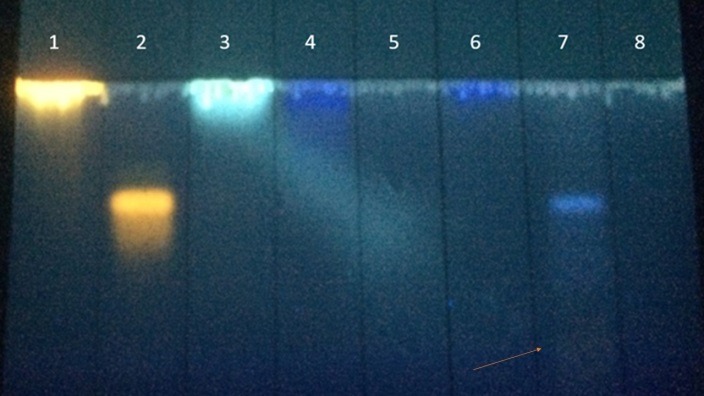
Thin layer chromatography (TLC) of phytochemical standards [quercetin (1), rutin (2), caffeic acid (3), tannic acid (4), coumarin (5), gallic acid (6), VhE (7) and solvent alone (8)]. Solvent: ethyl acetate, formic acid, acetic acid and water (100:11:11:27, respectively). Stain: NP – diphenylboric acid 2-aminoethyl ester, PEG – polyethylene glycol 4000. Arrow: yellow spot weekly revealed indicating an eventual presence of flavonoids in VhE.

*V. haenkeana* contains secondary metabolites such as tannins, saponins, phenolic compounds, flavonoids and coumarins.^21^ Here, we selected a sample *V. haenkeana* extract (VhE) to be subjected to Thin Layer Chromatography which revealed the presence of phenolic compounds characterized by a blue spot not matched with caffeic, tannic nor gallic acids; a weak yellow spot (indicated by an arrow) can be also seen in the chromatogram profile suggesting the presence of flavonoids not related to quercetin or rutin (Figure 1).

We have not observed the presence of coumarin in VhE under TLC system; our data were inconclusive to justify the involvement of this compound eventually present in VhE with treatment of respiratory diseases, as suggested by popular practices.^19,20^ However, it has already shown that commercial coumarin alone does not protect the neuromuscular blockade induced by *B. jararacussu* or *Crotalus durissus terrificus* venoms *in vitro.*^30^

Orange and blue compounds visualized by TLC in plant extracts are suggestive of flavonoids and polyphenol compounds. Oshima-Franco and Dal Belo^31^ reviewed antineurotoxic polyphenol plants, such as: *Camellia sinensis* L., *Casearia sylvestris* Sw., *Casearia gossypiosperma* Briquet, *Curcuma zedoaroides* A. Chaveerach & T. Tanee, *Dipteryx alata* Vogel, *Galactia glaucescens* Kunth*., Hypericum brasiliense* Choisy, *Jatropha elliptica* (Pohl) Oken*., Mikania laevigata* Sch. Bip. ex Baker*, Plathymenia reticulata* Benth*., Terminalia fagifolia* Mart., and *Vellozia flavicans* Mart. ex Schult. As showed in this work, *V. haenkeana* also has *in vitro* antineurotoxic effect, by an *in vitro* interaction.

*Vochysia haenkeana* has been poorly studied and its pharmacological properties are still unknown and need to be further explored. It has been shown that VhE does exhibit antitumoral activity in rats with induced Erlich tumor.^18^ In addition, our data have shown that VhE also does not promote antimicrobial action against *S. aureus*, *E. coli* and *P. aeruginosa* strains. Investigations about antimicrobial activities of plant extracts associated to local effects induced by snake venoms show potentially useful to indicate alternative methods to treat snakebites since infections caused by bacteria from snake’s mouth is often associated to snakebites.^32^

**Figure 2:**
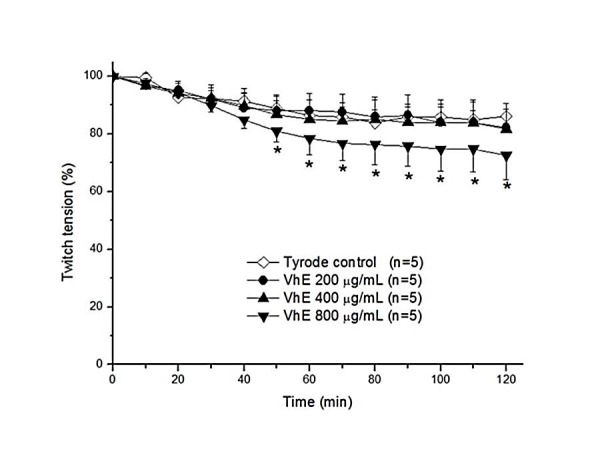
Neuromuscular responses of VhE (0.2–0.8 mg/mL) on mouse phrenic nerve-diaphragm (PND) preparation maintained under indirect stimulation. The points are the mean ± SEM (n = 5). *p < 0.05 compared to Tyrode control. VhE: *Vochysia haenkeana* extract.

Before subjecting VhE to neutralization assays in PND preparations, we have verified whether the extract by itself could cause changes in twitch responses; a concentration-response experiment was carried out using 0.2, 0.4 and 0.8 mg of VhE/mL; the concentrations of 0.2 and 0.4 mg/mL did not cause changes in PND preparations during 120 min incubation (*p* > 0.05) whereas 0.8 mg of VhE/mL induced a slight decrease in twitch tension from 50 min incubation (*p* < 0.05 compared to Tyrode solution alone). We have selected the VhE concentration of 0.4 mg/mL to be used in those protocols with neutralization of BthTX-I-induced neuromuscular blockade (50 µg/mL; concentration enough to produce complete neuromuscular blockade in PND preparations) (Figure 2).

**Figure 3:**
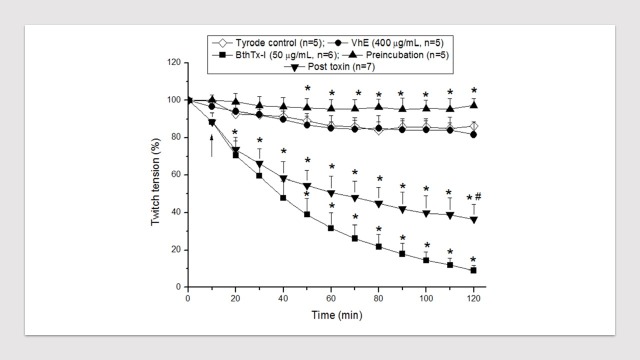
Effect of VhE on the neuromuscular blockade induced by BthTX-I on mouse phrenic nerve-diaphragm (PND) preparations maintained under indirect stimulation. Note that VhE prevented the BthTX-I blockade-induced under pre-toxin incubation. The points are the mean ± SEM (n = 5–7). *p < 0.05 compared to Tyrode control. ^#^*p* < 0.05 compared to BthTX-I alone. BthTX-I: Bothropstoxin-I; VhE: *Vochysia haenkeana* extract. Arrow indicates the exact moment in which VhE was added for post-toxin incubation protocols.

BthTX-I induces irreversible neuromuscular blockade and myonecrosis *in* vitro similarly to effects caused by crude venom of *Bothrops jararacussu* from where it comes from.^16,17^ The ability of this toxin to reproduce the neuromuscular effects seen with crude venom becomes it the main myotoxin from *B. jararacussu* venom*.* We have applied two different protocols to assess the neutralizing ability of VhE: pre-toxin incubation (BthTX-I was maintained under incubation for 30 min with VhE prior experiments in PND preparations) and post-toxin incubation (VhE was added into the record chamber 10 min after toxin addition). Under pre-toxin incubation, VhE neutralized completely the neuromuscular blockade caused by BthTX-I; on the other hand, VhE was not able to avoid the blockade by BthTX-I when added after toxin although it has been noticed a slight attenuation over 120 min (Figure 3).

Oshima-Franco et al.^15^ studied the presynaptic nature of BthTX-I. Thus, the preincubation of toxin with VhE observed in pre-toxin experiments can be related to ability of VhE to avoid the presynaptic trigger of myotoxin; which, in turn, once triggered VhE was unable of repairing (post-toxin experiments).

Table 1 compares the level of myotoxicity induced BthTX-I to those ones reached by VhE under pre- and post-toxin incubation protocols. The pool of BthTX-I used in this work caused low myotoxicity when compared to other data shown in previous investigations, where 20 μg of toxin/mL induced 67 ± 2.3 % of damage cell in PND preparations.^33^ Here, we have used 50 μg of toxin/mL to obtain complete muscle paralysis. Under pre- and post-toxin incubation protocols, VhE contributed to decrease in ~50 % the cell damage caused by BthTX-I.

**Table 1:** Morphological analysis of diaphragm muscle expressed as Myotoxicity Index (MI, in %)

**Muscles resulting from Pharmacological assays**	**MI (%)**
Tyrode control	8.04 ± 5
VhE	7.85 ± 5
BthTX-I	26.7 ± 16
Pre-toxin incubation	14.4 ± 10
Post-toxin incubation	14.8 ± 11

**Figure 4:**
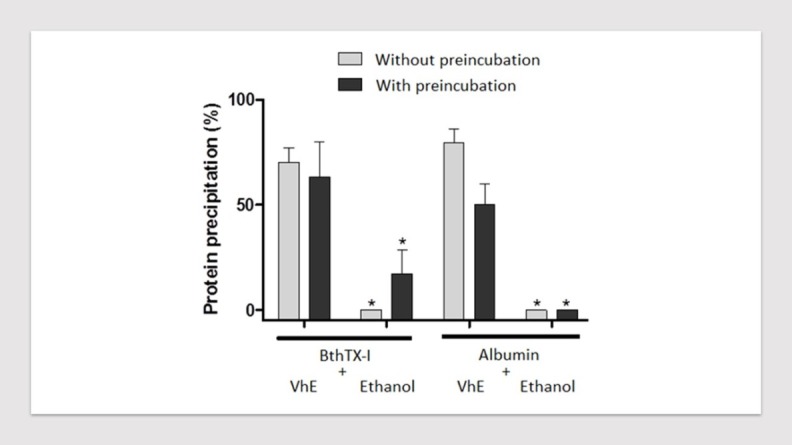
Protein precipitation induced by VhE. Note that VhE caused significant precipitation of BthTX-I and albumin; ethanol (used for solubilizing the VhE) had no activity on these effects. *p < 0.05. VhE: *V. haenkeana* extract.

The neutralizing ability of VhE against the neuromuscular blockade caused by BthTX-I *in vitro* may be related to its capacity to induced protein aggregation as seen in our experiments in protein precipitation assay (Figure 4). The incubation with VhE promoted the precipitation of BthTX-I reducing the toxin-concentration in 70.5 ± 7.1 %. The incubation of VhE with albumin has also been evaluated as positive control showing 79.4 ± 6.7 % of precipitation. Ethanol (solvent for VhE) alone was assessed under protein precipitation assay as negative control in order to refute its influence in that protein aggregation seen with VhE; it did not promote protein aggregation in both of compounds albumin and BthTX-I.

The pharmacological action of plant extracts to neutralize the neuromuscular activity of snake venoms and their toxins *in vitro* shows still unknown but it is frequently associated with protein precipitation, proteolytic degradation, enzyme inactivation, metal chelation and antioxidant action.^30^ Flavonoids and tannins are the main components related with those activities and both of them were previously observed in VhE.^21,27,34^ Although we have not found evidence for tannic acid in this extract, responsible for antiophidian mechanism of plant extracts as suggested by Melo et al.,^30^ the protein precipitation promoted by VhE may be related to its neutralizing effect against BthTX-I.

## Conclusion

*V. haenkeana* stem barks extract abolished the neuromuscular blockade caused by BthTX-I under pre-toxin incubation treatment; however, it was not efficient to neutralize the toxin-induced neuromuscular blockade under post-toxin treatment. In both of treatments VhE contributed to decrease the myotoxicity caused by toxin. These effects may be related to protein aggregation involving phenolic compounds which represent the major constituent of VhE, as shown by TLC. VhE showed no antimicrobial activity against *S. aureus*, *E. coli* and *P. aeruginosa* strains.

## Acknowledgments

This work was supported by Conselho Nacional de Desenvolvimento Científico e Tecnológico (Pibic/CNPq, Brazil,) and Fundação de Amparo à Pesquisa do Estado de São Paulo (FAPESP, Brazil, grant numbers: 04/09705-8; 07/53883-6; 08/52643-4; 12/08271-0; 15/01420-9). CH was supported by scientific initiation scholarship from Santander in partnership with Paulista University (UNIP, Brazil).

## Ethical Issues

This study was approved by the institutional Ethics Committee on Animal Use (CEUA/UNISO, protocol no. 054/2015) and the experiments were carried out according to the guidelines established by the Brazilian Society of Laboratory Animal Science (SBCAL).

## Conflict of Interest

The authors declare that they have no competing interests.
